# The sex difference in gait speed among older adults: how do sociodemographic, lifestyle, social and health determinants contribute?

**DOI:** 10.1186/s12877-021-02279-7

**Published:** 2021-06-02

**Authors:** Lena D. Sialino, Laura A. Schaap, Sandra H. van Oostrom, H. Susan J. Picavet, Johannes W.R. Twisk, W. M. Monique Verschuren, Marjolein Visser, Hanneke A.H. Wijnhoven

**Affiliations:** 1 Department of Health Sciences, Faculty of Science, Amsterdam Public Health research institute, Vrije Universiteit Amsterdam, Amsterdam, the Netherlands; 2 Centre for Nutrition, Prevention and Health Services, National Institute for Public Health and the Environment, Bilthoven, the Netherlands; 3 Department of Clinical Epidemiology and Biostatistics, Amsterdam University Medical Centre, Amsterdam, the Netherlands; 4 Julius Centre for Health Sciences and Primary Care, University Medical Centre, Utrecht, The Netherlands

**Keywords:** Physical aging, Risk factors

## Abstract

**Background:**

This study explores whether a sex difference in sensitivity to (strength of the association) and/or in exposure to (prevalence) determinants of gait speed contributes to the observed lower gait speed among older women compared to men.

**Methods:**

Data from the Longitudinal Aging Study Amsterdam (LASA) were used. In total 2407 men and women aged 55–81 years were included, with baseline measurements in 1992/2002 and follow-up measurements every 3–4 years for 15/25 years. Multivariable mixed model analysis was used to investigate sex differences in sensitivity (interaction term with sex) and in exposure to (change of the sex difference when adjusted) socio-demographic, lifestyle, social and health determinants of gait speed.

**Results:**

Women had a 0.054 m/s (95 % CI: 0.076 − 0.033, adjusted for height and age) lower mean gait speed compared to men. In general, men and women had similar determinants of gait speed. However, higher BMI and lower physical activity were more strongly associated with lower gait speed in women compared to men (i.e. higher sensitivity). More often having a lower educational level, living alone and having more chronic diseases, pain and depressive symptoms among women compared to men also contributed to observed lower gait speed in women (i.e. higher exposure). In contrast, men more often being a smoker, having a lower physical activity and a smaller personal network size compared to women contributed to a lower gait speed among men (i.e. higher exposure).

**Conclusions:**

Both a higher sensitivity and higher exposure to determinants of gait speed among women compared to men contributes to the observed lower gait speed among older women. The identified (modifiable) contributing factors should be taken into account when developing prevention and/or treatment strategies aimed to enhance healthy physical aging. This might require a sex-specific approach in both research and clinical practice, which is currently often lacking.

**Supplementary Information:**

The online version contains supplementary material available at 10.1186/s12877-021-02279-7.

## Background

Adequate gait speed is an important determinant of healthy physical aging, since it is protective of hospitalization, functional dependence, poor quality of life, institutionalization, disability and death in middle and old ages [1, 2]. Older women score lower on gait speed compared to older men [3]. This (height-adjusted) sex difference in gait speed among older adults has been well-established by multiple studies [3–6], but is not well understood. Previous research showed that sex differences in both sensitivity and exposure to mental, physical, social and lifestyle determinants can partly explain inequalities in general physical health between adult men and women [7]. The current study examines whether these two theories explain the observed lower gait speed among women compared to men among adults aged 55 years and older.

The sensitivity theory hypothesizes a difference between men and women in their vulnerability for the impact of determinants associated with physical health [7]. For example, one study showed that among older women the association between physical activity and gait speed was stronger compared to older men [8], so a decrease in physical activity may have a higher impact on gait speed decline in women. Moreover, sex-specific regression models show different lifestyle determinants of physical performance (including gait speed) for older men and women; namely doing exercise and engagement in social activities for men and sleep quality for women [9]. This suggests a sex difference (women versus men) in sensitivity towards these lifestyle factors, although interaction with sex was not statistically tested. Also, most studies simply adjust their multivariate regression models on determinants of physical performance for sex [10–12], limiting our knowledge on sex differences in sensitivity.

The exposure theory relates to a difference in exposure to determinants associated with physical health between men and women [7]. For example, older women are more often overweight compared to older men [13], so they are more exposed to overweight as a determinant of lower gait speed [14]. It is well known that older women in western societies smoke and drink less, are less physically active related to leisure and work time, are more often overweight, experience more sleep problems, are lower educated, experience more chronic diseases, report lower mental health and have more social connections compared to older men [7, 9, 15], which are all known determinants of physical performance [9, 16]. Some of these factors may protect women more compared to men, such as less smoking, while other factors, like more often being obese, may contribute to the observed lower gait speed among women compared to men. This has, however, not been studied before. An American cohort study demonstrated that sex differences in body composition measures (such as higher total body fat among older women), partly explained the sex difference in physical performance (including gait speed), while the higher prevalence of chronic diseases in women did not [17]. However, these results were based on univariable models and limited in the number of determinants tested.

Exploring contributing (modifiable) determinants for the observed lower gait speed among older women compared to men will provide important information that prevention strategies aimed to enhance healthy physical aging should take into account. Therefore, the current study investigates whether differences between men and women in sensitivity and/or exposure to various sociodemographic, lifestyle, social and health determinants of lower gait speed contribute to the observed lower gait speed among women in adults aged 55 years and older.

## Methods

### Analytical sample

Data from the prospective Longitudinal Aging Study Amsterdam (LASA) were used, which contains a nationally representative sample from three culturally distinct regions in the Netherlands (Amsterdam, Zwolle and Oss) [18]. Measurements and interviews were performed by trained interviewers, who visited respondents at home. Longitudinal data of two birth cohorts were combined, with baseline measurements in 1992/1993 (*n* = 3107, aged 55–81, 25 year follow-up) and in 2002/2003 (*n* = 1002, aged 55–65, 15 year follow up). Follow-up measurements were performed every three to four years. The multivariable analysis included participants with complete data on all determinants per measurement, resulting in 2407 participants in our analytical sample with an average of 2.6 measurements per participant (6244 observations in total). Response rates were high and drop-out was low; approximately 3 % per follow-up measurement, and mainly due to mortality [19]. Ethical approval for the LASA study was given by the Medical Ethics Committee of the VU University Medical Centre Amsterdam, and all participants provided written informed consent.

### Gait speed

Gait speed was measured by asking participants to walk 3 m, turn around and walk 3 m back as quickly as possible [19, 20]. The distance was indicated on the ground by measurement tape and trained staff recorded the total time needed using a stopwatch. Gait speed is expressed in meters per second.

### Socio-demographic determinants

Education was categorized into low (elementary education or less), middle (lower vocational education and general intermediate education) and high education (intermediate vocational education, general secondary education, higher vocational education, college education and university) [4]. Age was calculated by the amount of days between the date of the interview and the birth date, expressed in years. Sex was defined as men or women and asked for at baseline.

### Lifestyle determinants

Alcohol consumption was defined as number of alcohol drinks per day, categorized into none, up to two and more than two. Smoking status was defined as never, former and current smoker. Physical activity was asked using the validated LASA Physical Activity Questionnaire (LAPAQ) and defined as total MET hours/week using the Dutch guidelines for physical activity [21], including walking outdoors, light household activities, heavy household activities and two most frequently performed sports performed in the past two weeks [22, 23]. Sleep problems were defined as problems with falling asleep, waking through the night and/or waking up too early in the morning, calculating the combined score ranging from 0 (no problems) to 9 (many problems). Sleep duration was defined as hours of sleep in 24 h, categorized into less than 7 h (short), between 7 and 9 h (normal) and more than 9 h (long). Body Mass Index (BMI) was calculated by dividing weight in kg by height in m squared, where weight was measured to the nearest 0.1 kg using a calibrated bathroom scale (Seca, model 100, Lameris, Utrecht, The Netherlands) and height was measured to the nearest 0.1 cm using a stadiometer. If these measurements were missing, self-reported weight (approximately 1 % of all measures) and height (approximately 2 % of baseline measures) were used.

### Social determinants

Personal network size was defined as the count of “the people with whom you are in touch regularly and who are important to you”, resulting in a continuous scale. Living situation was defined as living alone or living with partner/spouse/others. Social participation was divided into formal and informal social participation. Formal social participation was defined as visiting 13 different types of organisations (such as trade union, political party, church, hobby club etc.) categorized into: does not visit up to few times a year; once up to few times a month; few times a week; and few times a week up to every day [24]. Informal social participation included six recreational trips (such as museum, social cultural centrum, restaurant etc.) with a frequency ranging from 0 (never) to 6 (every day), calculated into a combined score ranging from 0 to 42 [24]. Loneliness was measured using the 11-item De Jong Gierveld scale ranging from 0 (no loneliness) to 11 (severe loneliness) [25].

### Health determinants

Self-reported chronic diseases was defined as the number of chronic diseases of the most frequently occurring somatic chronic disease in the Netherlands: chronic non-specific lung disease, cardiac disease, peripheral artery diseases, diabetes mellitus, cerebrovascular accident or stroke, osteoarthritis, rheumatoid arthritis and/or cancer and a maximum of two other chronic diseases which symptoms lasted for at least three months. The total score ranged from 0 to 3 (three or more chronic diseases). Pain during the past week was assessed by a 6-item yes/no subscale based on the Nottingham Health Profile, ranging from 0 (no pain) to 5 (much pain) [26]. Pain was categorized into no (0), little (1), some (2) and much pain (3–5). Depressive symptoms during the past week were assessed using the 20-item Centre for Epidemiologic Studies Depression Scale (CES-D), ranging from 0 (rarely or none of the time; less than one day per week) to 3 (most or almost all the time; five–seven days per week), transformed into a score ranging from 0 to 60 [27].

### Statistical analyses

Mixed model analyses for repeated measures with gait speed as dependent variable were used to investigate sex differences in sensitivity and exposure to determinants of gait speed. Gait speed and all determinants were time-varying (except for height, educational level and sex). To elaborate: In the mixed model analyses, the analyses are performed per measurement wave in cross-sectional nature, were after the results are pooled with adjustments for repeated measures in the individual. All models were adjusted for age, birth cohort and baseline height. The full multivariable model further included all determinants.

The sensitivity theory hypothesizes that the strength of the association between the determinant and gait speed differs between men and women. This was investigated by first analysing the full multivariable model (including all determinants) for men and women separately. Thereafter, each interaction term (determinant*sex) was added separately to a full multivariable model combining men and women and tested for significance (p < 0.10).

The exposure theory hypothesizes that the difference in prevalence of (i.e. exposure to) determinants of gait speed between men and women contributes to observed the sex difference. This was investigated by testing the impact of adjusting for each individual determinant on the regression coefficient of the variable “sex”, while adjusted for all other determinants. This method is commonly used to assess confounding. In the current study, the impact of this determinant on the sex difference in the outcome is assessed. A meaningful impact was set beforehand at a change of the regression coefficient “sex” (denoting the sex difference in gait speed) by > 5 %, as is commonly used for confounding.

## Results

### Analytical sample

At baseline, the analytical sample (n = 2407) had a similar number of men and women, with a similar mean age. Compared to men, women at baseline were lower educated, less tall, had a lower gait speed, a healthier lifestyle regarding alcohol consumption, smoking and physical activity, but had a higher mean BMI, more sleep problems, more often lived alone, had more chronic diseases, more often pain and reported more depressive symptoms (Table 1). When compared to the total LASA study sample (Table S1), the analytical sample had similar baseline characteristics.

**Table 1:** Baseline characteristics of the analytical sample (n = 2407) for men and women

Characteristics	Men (50 %)	Women (50 %)
**Socio-demographic**		
Age (years)	66.5 (0.24)	65.6 (0.22)
Education	-	-
Low	22.9	36.5
Middle	32.0	38.2
High	45.1	25.3
Height (m)	1.75 (0.00)	1.63 (0.00)
**Physical performance**		
Gait speed (m/s)	0.93 (0.01)	0.88 (0.01)
**Lifestyle**		
Alcohol consumption	-	-
None	9.57	20.0
Light/moderate (up to 2 per day)	57.1	65.9
Heavy/extreme (more than 2 per day)	33.3	14.1
Smoking status	-	-
Never	10.5	48.3
Former	58.9	34.9
Current	30.7	16.8
Physical activity (MET hours per week) ^a^	57.0 (1.31)	74.8 (1.23)
Sleep problems (ranging from 0–9) ^b^	5.20 (0.06)	6.10 (0.06)
Sleep duration (per 24 h)	-	-
Short (less than 7 h)	14.3	20.4
Normal (between 7 and 9 h)	68.3	65.6
Long (more than 109 h)	17.4	14.0
BMI (kg/m^2^)	26.4 (0.09)	27.4 (0.13)
**Social**		
Personal network size (range 0–80) ^c^	15.2 (0.26)	15.8 (0.25)
Living situation (not living alone)	84.0	63.3
Social participation formal ^d^	-	-
Up to few times a year	35.4	23.8
Few times a year up to few times a month	20.8	20.4
Few times a month up to every week	21.3	27.3
Every week up to every day	22.5	28.5
Social participation informal (range 0–42) ^e^	9.55 (0.15)	9.71 (0.14)
Loneliness (range 0–11)	1.59 (0.06)	1.81 (0.07)
**Health**		
Chronic diseases (self-reported)	-	-
None	31.1	25.6
One	35.7	33.2
Two	20.5	23.8
Three or more	12.7	17.4
Pain (in the past week)	-	-
No	76.8	66.5
Little	9.62	12.8
Some	5.54	6.73
Much	8.07	13.9
Depressive symptoms (CES-D, range 0–60)	5.67 (0.17)	8.32 (0.22)

Note: Explanation: mean (sd) or percentage (%)

^a^ Including walking outdoors, light and heavy household activities and two most frequency performed sports

^b^ Combined score of ever having problems with falling asleep, waking through the night and too early

^c^ Count of people with whom you are in contact regularly and are important to you

^d^ Visiting 13 different types of organizations (such as trade union, political party, church, hobby club etc.)

^e^ Combined score of 6 recreational trips (museum, restaurant etc.) with a frequency from never to every day

### The sex difference in gait speed

**Fig. 1:**
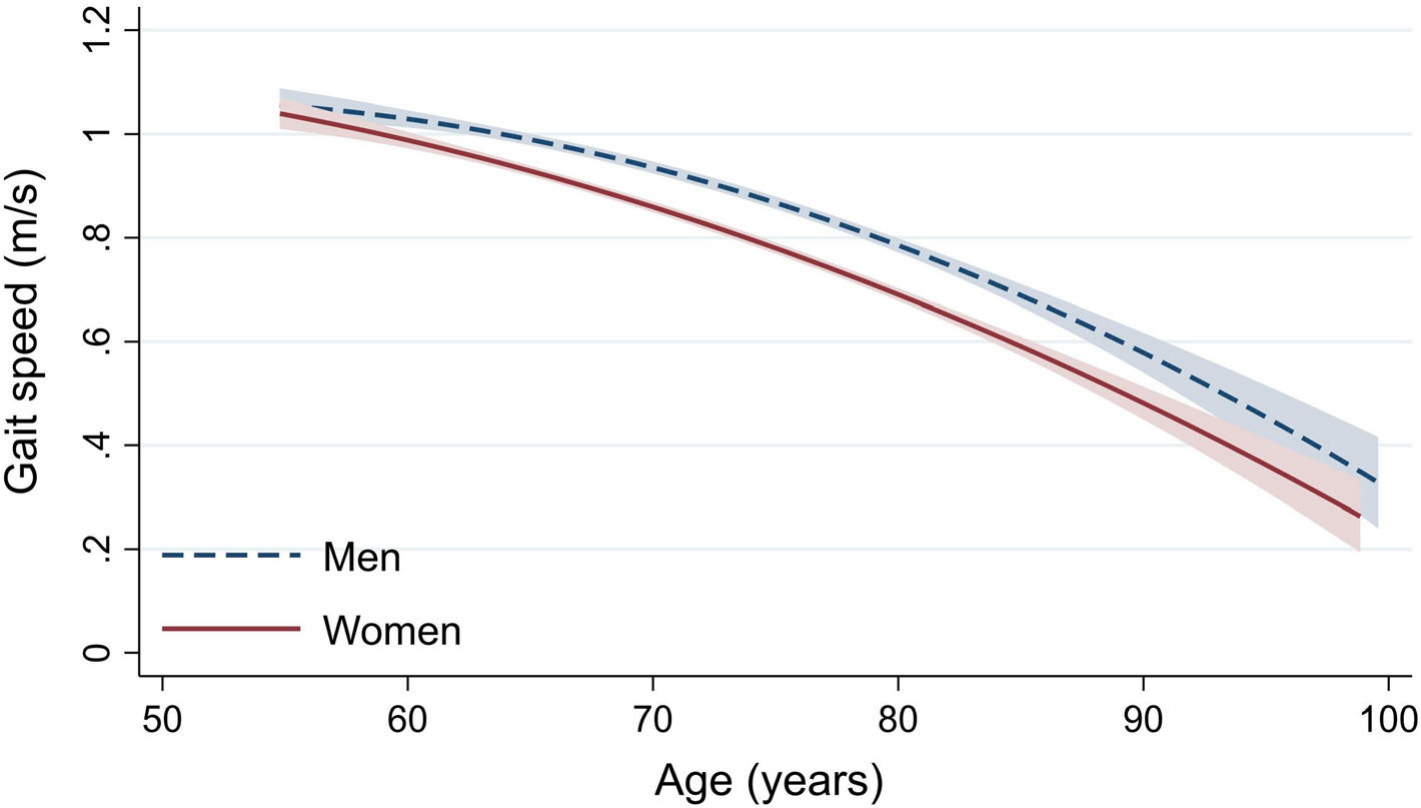
Unadjusted fitted line of gait speed with 95 % confidence interval for men (blue dashed line) and women (red solid line) by age of the analytical sample (*n* = 2407)

There was a consistent sex difference in gait speed among adults aged 55 years and older, in which women walked slower compared to men (0.076 m/s 95 % CI: 0.057–0.094, *p* < 0.001, unadjusted). The sex difference remained relatively stable by age (Fig. 1). When adjusted for baseline height, age and birth cohort, women still had a 0.054 m/s (95 % CI: 0.033–0.076, *p* < 0.001) lower mean gait speed compared to men.

### Sensitivity theory

Men and women had similar determinants of gait speed (adjusted for age and baseline height) in a multivariable model, in which lower educational level, alcohol abstinence, less social participation, having three or more chronic diseases and more pain and depressive symptoms were statistically significantly associated with lower gait speed (Table 2). However, in women, lower physical activity and higher BMI were both more strongly associated with lower gait speed compared to men (*P* interaction determinant*sex < 0.10). To illustrate: the association of higher BMI with lower gait speed was stronger for women compared to men (*P* interaction = 0.019); one unit higher BMI was associated with a 0.006 m/s lower gait speed (95 % CI: 0.008 − 0.004) in women and a 0.001 m/s lower gait speed in men (95 % CI: -0.005–0.02). There were also sex differences in the association between pain and gait speed, but this was an inconsistent finding: Men were more sensitive to “little” versus “no pain”, women were more sensitive to “some” versus “no pain” and there was no sex difference in sensitivity to “much” versus “no pain”. Although men and women differed in their association of sleep problems and sleep hours (short versus normal) with gait speed (*P* interaction < 0.10), both determinants were not associated with gait speed for men or women.

**Table 2:** Sex differential sensitivity: sex stratified multivariable association model with gait speed (m/s) as dependent variable

	Multivariable model with gait speed (m/s) as dependent variable ^a^				
	**Men**		**Women**		**Interaction**^**b**^
Determinants	Beta	[95 % CI]	Beta	[95 % CI]	*P*-value
**Socio-demographic**					
Education				-	-
Middle vs. low	**0.033**	[ 0.004 – 0.063]	**0.031**	[ 0.008 – 0.054]	0.94
High vs. low	**0.068**	[ 0.040 – 0.097]	**0.053**	[ 0.027 – 0.080]	0.49
**Lifestyle**					
Alcohol consumption				-	-
Max 2 per day vs. never	**0.050**	[ 0.017 – 0.083]	0.013	[-0.008 – 0.034]	0.11
2 + per day vs. never	**0.039**	[ 0.003 – 0.075]	**0.032**	[ 0.001 – 0.063]	0.73
Smoking status				-	-
Former vs. never	-0.003	[-0.035 – 0.029]	0.008	[-0.012 – 0.028]	0.73
Current vs. never	-0.019	[-0.056 – 0.018]	**-0.048**	[-0.079 – − 0.017]	0.36
Physical activity (MET hours/week)	0.0002	[-0.000 – 0.000]	**0.0004**	[ 0.000 – 0.001]	**0.02**
Sleep problems (range 0–9)	0.0004	[-0.005 – 0.006]	0.005	[-0.000 – 0.009]	**0.03**
Sleep duration				-	-
Short vs. normal	-0.015	[-0.043 – 0.014]	0.015	[-0.006 – 0.036]	**0.02**
Long vs. normal	0.013	[-0.011 – 0.037]	0.002	[-0.023 – 0.026]	0.40
BMI (kg/m2)	-0.001	[-0.005 – 0.002]	**-0.006**	[-0.008 – − 0.004]	**0.02**
**Social**					
Personal network size (n)	**0.002**	[ 0.001 – 0.003]	0.001	[-0.000 – 0.002]	0.11
Living situation (not alone vs. alone)	0.022	[-0.004 – 0.049]	0.001	[-0.019 – 0.020]	0.38
Social participation formal	-	-	-	-	-
Up to few times a month vs few/year	0.012	[-0.012 – 0.036]	0.012	[-0.011 – 0.034]	0.82
Every week vs. few/year	-0.009	[-0.032 – 0.015]	0.012	[-0.009 – 0.034]	0.27
Every week up to every …day vs. few/year	**0.053**	[ 0.030 – 0.077]	**0.034**	[ 0.012 – 0.056]	0.30
Social participation informal (range 0–42)	**0.003**	[ 0.001 – 0.005]	**0.005**	[ 0.003 – 0.007]	0.13
Loneliness (Gierveld, range 0–11)	0.002	[-0.003 – 0.006]	-0.004	[-0.007 – 0.000]	0.55
**Health**					
Chronic diseases				-	-
One vs. none	0.006	[-0.019 – 0.030]	-0.013	[-0.037 – 0.011]	0.19
Two vs. none	-0.010	[-0.036 – 0.017]	-0.013	[-0.039 – 0.014]	0.61
Three or more vs. none	**-0.035**	[-0.065 – 0.005]	**-0.034**	[-0.062 – − 0.006]	0.52
Pain					
Little vs. no	**-0.040**	[-0.067 – − 0.012]	-0.005	[-0.027 – 0.018]	**0.07**
Some vs. no	**-0.039**	[-0.077 – − 0.002]	**-0.077**	[-0.106 – − 0.049]	**0.07**
Much vs. no	**-0.115**	[-0.151 – − 0.078]	**-0.111**	[-0.135 – − 0.086]	0.99
Depressive symptoms (CES-D, range 0–60)	**-0.004**	[-0.006 – − 0.002]	**-0.003**	[-0.004 – − 0.002]	0.22

Note: bold = significant (beta *p*<0.10)

Note: To illustrate: The association of the determinant BMI with gait speed is stronger for older women compared to older men (*p*=0.02) in a multivariable model. An increase of one unit BMI (kg/m2) is associated with a decrease in gait speed of 0.006 m/s (95 % CI: -0.008 ― -0.004) in older women and of 0.001 m/s (95 % CI: -0.005 - 0.006) in older men.

^a^ Includes birth cohort, age and baseline height and all determinants (column one)

^b^ All interactions of sex*determinant (indicated by row) are individually tested in the full model

### Exposure theory

To test the exposure theory, the contribution of each determinant to the sex difference in gait speed was examined while adjusting for all other determinants in a multivariate model adjusted for age and baseline height. The sex difference in gait speed significantly decreased (> 5 %) when adjusted for educational level, living situation, chronic diseases, pain or depressive symptoms (Table 3). So, for women, more often being lower educated, more often living alone and having a higher prevalence of chronic disease, pain and depressive symptoms compared to men, contributed to the lower gait speed among women compared to men. To illustrate: Adjusting for pain in a multivariable model resulted in a decrease of the regression coefficient denoting the multivariate adjusted sex difference in gait speed from − 0.037 to -0.029. So, pain contributed (1 - (-0.037 / -0.029)) 25.4 % to the observed sex difference in gait speed. Note that the values in the tables are rounded off. In contrast, the sex difference in gait speed significantly increased (> 5 %) when adjusted for smoking status, physical activity or personal network size in a multivariable model (Table 3). So, men more often being a smoker, having a smaller personal network size and being less physically active compared to women decreases the gait speed among men. Consequently, it does not contribute but suppresses the observed lower gait speed among women compared to men.

**Table 3:** Sex differential exposure: impacting the sex difference in gait speed in a multivariable association model

	Beta “sex” [95% CI] ^b^	Percentage change ^c^
Full model ^a^	**− 0.029** [-0.051 ― − 0.008]	Reference
**Socio-demographic**		
Education (high/middle versus low)	**− 0.032** [-0.054 ― − 0.011]	**- 10.5 %**
**Lifestyle**		
Alcohol consumption (vs. never)	**− 0.030** [-0.051 ― − 0.008]	- 0.7 %
Smoking status (vs. never)	**− 0.028** [-0.048 ― − 0.007]	**+ 6.3 %**
Physical activity (MET hours / week)	**− 0.027** [-0.048 ― − 0.006]	**+ 8.4 %**
Sleep problems (range 0–9)	**− 0.028** [-0.050 ― − 0.007]	+ 4.5 %
Sleep duration (short/long vs. normal)	**− 0.030** [-0.051 ― − 0.008]	- 0.9 %
BMI (kg/m2)	**− 0.030** [-0.052 ― − 0.009]	**-** 3.4 %
**Social**		
Personal network size (n)	**− 0.026** [-0.048 ― − 0.005]	**+ 10.8 %**
Living situation (alone vs. not alone)	**− 0.033** [-0.054 ― − 0.012]	**- 12.9 %**
Social participation formal (vs. few a year)	**− 0.029** [-0.050 ― − 0.007]	+ 2.1 %
Social participation informal (range 0–42)	**− 0.028** [-0.050 ― − 0.007]	+ 3.0 %
Loneliness (Gierveld scale, range 0–11)	**− 0.029** [-0.051 ― − 0.008]	+ 1.1 %
**Health**		
Chronic diseases (vs. none)	**− 0.031** [-0.053 ― − 0.010]	**- 6.4 %**
Pain (vs. no)	**− 0.037** [-0.059 ― − 0.015]	**- 25.4 %**
Depressive symptoms (CES-D, range 0–60)	**− 0.033** [-0.055 ― − 0.012]	**- 10.0 %**

Note: Each row represents the model excluding the indicated determinant

Note: Bold = significant (beta *p*<0.05, percentage change >5.0 %)

Note: A negative percentage change represents a contributing determinant (the sex difference decreases when adjusted for this determinant) and a positive percentage change represents a suppressing determinant (the sex difference increases)

Note: To illustrate: Adjusting for pain in a multivariable model causes a decrease of the sex difference (from -.0037 to -0.029). So, pain contributes (25.4 %) to the observed sex difference in gait speed.

^a^ Full model includes birth cohort, age, baseline height and all determinants (column one)

^b^ Represents the regression coefficient for the association between sex (women vs men) and gait speed

^c^ Percentage change is 1 - (Beta “sex” model excluding the indicated determinant) / Beta “sex” full model, representing the change in the sex difference in gait speed when adjusting for the indicated determinant

## Discussion

The findings of this study show that both the sex differential sensitivity to lower physical activity and higher BMI, and sex differential exposure to lower educational level, living alone, chronic diseases, pain and depressive symptoms contribute to the observed lower gait speed among women aged 55 years and older compared to men.

The observed lower gait speed among women aged 55 years and older (0.054 m/s, adjusted for age and height) is in line with previous research demonstrating a sex difference in older adults ranging from − 0.03 to -0.10 m/s across different countries [3, 4, 6]. The studies demonstrating a greater sex difference than the current study did not adjust for body height, which likely caused an overestimation of the sex difference [28]. Adjusting for body height adjusts for the biological advantage of men being taller and therefore having a higher gait speed [28]. Since the current study focused on the sex difference in gait speed caused by modifiable determinants, all analyses were adjusted for height.

The sex difference seems to represent a clinically relevant difference since it equalizes the minimal clinically significant individual difference estimates for gait speed, ranging between 0.03 and 0.05 m/s [5]. In addition, gait speed cut-offs among well-functioning (older) adults related to various health-related events are similar for men and women [1]. So, the sex difference in gait speed may represent a female disadvantage in healthy aging [3]. However, additional research regarding the observed sex difference and its association with negative health outcomes is needed. After adjustment for all determinants in the current multivariable analyses, the observed lower gait speed among women decreased to 0.03 m/s. This may imply that there is a remaining biological difference and/or that other contributing determinants are missing from the current study, such as gender or dietary habits. Although beyond the scope of this study, other potential biological determinants have been proposed to account for sex differences in physical performance, such as sex hormones [29], body composition [17, 30] and physiological differences in muscle function and structure [30]. These may explain the unexplained part of the sex difference in gait speed in the current study and could also explain the contributing role of some variables in our model like chronic diseases. Further research is needed to further explain these mechanisms.

In general, men and women have similar determinants of lower gait speed. However, based on the current findings, women aged 55 years and older seem more sensitive for the negative impact of lower physical activity and higher BMI compared to men (sensitivity theory). This is in accordance with findings from previous studies showing that lower physical activity and higher BMI are both more strongly associated with lower gait speed among older women, by comparing sex-stratified models (without testing for statistical interaction) [8, 14, 31]. Although one study found a stronger association between higher BMI and lower gait speed for older men, this finding was based on very few men with high and low BMI in an univariable model only [30].

As physical activity, BMI and pain are inter-correlated [32–34], a multifactorial pathway may exist. For example, there are various mechanisms for obesity-related pain and pain can also lead to an increase in BMI through a physically inactive lifestyle among older adults [32, 35]. Also, most (older) women have a higher percentage body fat compared to men, which is associated with higher BMI and lower physical activity (and even stronger association for older women compared to men) [17, 36, 37]. So, higher body fat percentage might drive the higher sensitivity towards BMI and physical activity in association with gait speed among older women. Lower muscle mass and strength among older women compared to men may also explain the stronger association between BMI and lower gait speed in women compared to men [38]. However, the individual associations of the determinants physical activity and BMI with gait speed remained significantly different between older men and women in a multivariable model. This suggests they also have a significant individual contributing role.

The current study demonstrated that women more often being lower educated, living alone and having chronic diseases, pain and depressive symptoms compared to men contributes to the lower observed gait speed among women compared to men (exposure theory). Tseng et al. demonstrated that the higher prevalence of chronic diseases among older women did not contribute to the sex difference in gait speed [17], while the current study and others [34] suggest it does contribute. The discrepancy with Tseng et al. might be due to their individual testing of chronic health conditions, which might limit the statistical contributing effect of chronic diseases overall. No previous studies investigated the contributing role of educational level, living alone, chronic diseases and depressive symptoms in association with objectively measured gait speed or other physical function tests. However, educational level and depressive symptoms have been demonstrated to explain part of the sex difference in self-reported physical disabilities in daily life [39]. Furthermore, educational level is known to influence physical health and gait speed among older adults by increasing a person’s ability to access health care and understand information on healthy lifestyle and the health care system [40]. In addition, pain, depressive symptoms and social isolation (related to living alone) are known to be inversely correlated with gait speed [41–43].

There are also three determinants of the sex difference in gait speed identified in the current study that contribute to a higher gait speed among women compared to men. Women more often have a “never” smoking status, a larger social network size and higher levels of physical activity compared to men, which are all positively associated with gait speed and various other measures of physical performance [16]. Despite the advantageous role of these determinants for women, they still on average had a significantly lower gait speed compared to men.

From a prevention point of view, the identification of modifiable determinants of gait speed in women including those that contribute to the sex difference, form an important starting point. Women take up an increasing percentage of our aging population due to a higher life expectancy, so they are an important target group for prevention aimed to enhance healthy physical aging [3]. Indeed, decreasing the modifiable lifestyle determinants BMI (when overweight) and increasing physical activity among older adults can improve their gait speed [44, 45]. Weight managing interventions might additionally decrease the observed lower gait speed among women compared to men, since our study and others suggests that older women are more sensitive to the impact of higher BMI and lower physical activity and are additionally more likely to become overweight and obese compared to men [13]. However, also older men gain weight while growing older, so it is still important to include both sexes in these interventions [46]. Note, weight loss strategies among older adults should be combined with sufficient protein intake and physical activity (in particular resistance training) to reduce the risk of sarcopenia and osteoporosis [47]. Although not immediately modifiable, reducing the sex differences in chronic diseases, depressive symptoms and especially pain might also decrease the observed lower gait speed among women compared to men. Therefore, prevention and treatment strategies need to take sex differences in disease course and treatment into account, which is often not the standard yet [48]. Furthermore, to date, the education gap between men and women is reducing [49], so in more recent cohorts its contributing role for the observed lower gait speed among women may diminish.

The current study has several strengths. First, data from a large prospective longitudinal cohort study were used, representing the older adult life course of the Dutch population. The longitudinal data allowed us to pool cross-sectional results across various measurement waves, increasing power and confidence of the current findings (2407 participants included, 6244 observations in total). To note, response rates were high and drop-out was low. Secondly, for the first time many different determinants from socio-demographic, lifestyle, social and health domains were individually tested in multivariable analyses. A limitation of the current study was the complete cases approach of mixed models, including only participants with complete data on the determinants on a single wave. Since baseline height was measured and asked in an additional medical interview, several participants were excluded (n = 682). Of the other determinants, especially social participation contained a lot of missing values, but the excluded participants also had missings across other determinants. Since the baseline characteristics and the sex difference (including its course) of our analytical sample were similar compared to the full LASA study population, it was assumed that these missings did not influence our conclusions. Also, the gait speed measurement used differs from the most common measures due to the turn. However, it was demonstrated that walking length and a turn or no turn does not influence the validity of the measurement [50, 51]. Another possible limitation of the current study is that gender was not taken into account (since it was not yet measured in LASA). In the IMIAS cohort of older adults it was demonstrated that feminine gender roles (tender, warm, affectionate, gentle, sympathetic and sensitive to other needs) are risk factors for mobility disability and low physical performance, independent of sex [52]. However, with regard to the development and implementation of prevention programs, it is more feasible to focus on sex (women and men) rather than gender (biological, cultural, societal and identity roles).

## Conclusions

The findings of this study show that both the sex differential sensitivity to higher BMI and lower physical activity and sex differential exposure to lower educational level, living alone, chronic diseases, pain and depressive symptoms contribute to the observed lower gait speed among older women aged 55 years and older compared to men. Women are more sensitive and/or exposed to these (modifiable) determinants associated with lower gait speed. To date, there is little emphasis on sex differences in preventive interventions or treatments aimed at healthy physical and functional aging, such as weight loss strategies and treatments for chronic conditions. Future research and intervention programs aiming to increase healthy physical aging among both older women and men should take sex differences in physical aging and its determinants into account.

## Supplementary Information

**Additional file 1:** Baseline characteristics of the LASA full study population for men and women.

## Data Availability

Data cannot be shared publicly because of confidentiality. Data are available from the LASA Institutional Data Access / Ethics Committee (contact via https://www.lasa-vu.nl/index.htm) for researchers who meet the criteria for access to confidential data. The data underlying the results presented in the study are available from the Longitudinal Aging Study Amsterdam (https://www.lasa-vu.nl/index.htm). The LASA Steering Group will review all requests for data to ensure that proposals for the use of LASA data do not violate privacy regulations and are in keeping with informed consent that is provided by all LASA participants. The authors of this study do not have any special access privileges to the data underlying this study that other researchers would not have.
