# Supplementary material for: The sex difference in gait speed among older adults: how do sociodemographic, lifestyle, social and health determinants contribute?
Source: BMC Geriatr. 2021 Jun 2;21:340. doi: 10.1186/s12877-021-02279-7 (PMC8173843; doi:10.1186/s12877-021-02279-7)
Supplement: Supplementary file 1 — Additional file 1: Baseline characteristics of the LASA full study population for men and women. [file 12877_2021_2279_MOESM1_ESM.docx]

**The sex difference in gait speed among older adults: how do sociodemographic, lifestyle, social and health determinants contribute?**

Lena D. Sialino MSc^1*^, Dr. Laura A. Schaap^1^, Dr. Sandra H. van Oostrom^2^, Dr. H. Susan J. Picavet^2^, Prof. Johannes W.R. Twisk^3^, Prof. W.M. Monique Verschuren^2,4^, Prof. Marjolein Visser^1^, Dr. Hanneke A.H. Wijnhoven^1^

^1^ Department of Health Sciences, Faculty of Science, Amsterdam Public Health research institute, Vrije Universiteit Amsterdam, Amsterdam, the Netherlands

^2^ Centre for Nutrition, Prevention and Health Services, National Institute for Public Health and the Environment, Bilthoven, the Netherlands

^3^ Department of Clinical Epidemiology and Biostatistics, Amsterdam University Medical Centre, the Netherlands

^4^ Julius Centre for Health Sciences and Primary Care, University Medical Centre, Utrecht, The Netherlands

* Corresponding author

**Table S1.** Baseline characteristics of the LASA full study population for men and women

|  | **LASA full sample (4109)** | |
| --- | --- | --- |
| Characteristics | Men (48%) | Women (52%) |
| **Socio-demographic** |  |  |
| Age (years) | 68.3 (0.20) | 68.0 (0.20) |
| Education | - | - |
| Low | 29.9 | 47.0 |
| Middle | 31.3 | 32.3 |
| High | 38.9 | 20.8 |
| Height (m) | 1.75 (0.00) | 1.62 (0.00) |
| **Physical performance** |  |  |
| Gait speed (m/s) | 0.90 (0.01) | 0.82 (0.01) |
| **Lifestyle** |  |  |
| Alcohol consumption | - | - |
| None | 12.7 | 24.3 |
| Light/moderate (up to 2 per day) | 53.6 | 62.7 |
| Heavy/extreme (more than 2 per day) | 33.7 | 13.0 |
| Smoking status | - | - |
| Never | 9.22 | 47.6 |
| Former | 56.9 | 32.9 |
| Current | 33.9 | 19.6 |
| Physical activity (MET hours per week) ^a^ | 55.4 (1.07) | 71.6 (0.99) |
| Sleep problems (ranging from 3-12) ^b^ | 5.31 (0.05) | 6.22 (0.05) |
| Sleep hours (per 24 hours) | - | - |
| Short (less than 7 hours) | 15.6 | 21.6 |
| Normal (between 7 and 9 hours) | 65.7 | 63.7 |
| Long (more than 10 hours) | 18.6 | 14.7 |
| BMI (kg/m^2^) | 26.3 (0.08) | 27.6 (0.11) |
| **Social** |  |  |
| Personal network size (range 0-80) ^c^ | 14.0 (0.20) | 14.4 (0.19) |
| Living situation (living with partner) | 78.6 | 66.8 |
| Social participation formal ^d^ | - | - |
| Up to few times a year | 37.4 | 26.7 |
| Few times a year up to few times a month | 19.1 | 19.4 |
| Few times a month up to every week | 21.5 | 26.5 |
| Every week up to every day | 22.1 | 27.4 |
| Social participation informal (range 0-42) ^e^ | 8.88 (0.12) | 8.72 (0.12) |
| Loneliness (range 0-11) | 1.92 (0.05) | 2.10 (0.06) |
| **Health** |  |  |
| Chronic diseases (self-reported) | - | - |
| None | 29.3 | 23.0 |
| One | 35.4 | 33.1 |
| Two | 20.2 | 23.7 |
| Three or more | 15.2 | 20.3 |
| Pain (in the past week) | - | - |
| None | 75.1 | 64.3 |
| Little | 9.80 | 14.0 |
| Some | 5.61 | 7.18 |
| Much | 9.54 | 14.6 |
| Depressive symptoms (CES-D, range 0-60) | 6.63 (0.15) | 9.25 (0.18) |

*Note:* Explanation: mean (sd) or percentage (%)

^a^ Including walking outdoors, light and heavy household activities and two most frequency performed sports

^b^ Combined score of ever having problems with falling asleep, waking through the night and too early

^c^ Count of people with whom you are in contact regularly and are important to you

^d^ Visiting 13 different types of organizations (such as trade union, political party, church, hobby club etc.)

^e^ Combined score of 6 recreational trips (museum, restaurant etc.) with a frequency from never to every day
